# Understanding Why Patients Return to the Emergency Department after Mild Traumatic Brain Injury within 72 Hours

**DOI:** 10.5811/westjem.2015.2.23546

**Published:** 2015-04-02

**Authors:** Latha Ganti, Lauren M. Conroy, Aakash Bodhit, Yasamin Daneshvar, Pratik Shashikant Patel, Sarah Ayala, Sudeep Kuchibhotla, Kelsey Hatchitt, Christa Pulvino, Keith R. Peters, Lawrence L. Lottenberg

**Affiliations:** * North Florida South Georgia Veterans Affairs Medical Center, Lake City, Florida; † University of Florida College of Medicine, Gainesville, Florida; ‡ Saint Louis University, Department of Neurology, Saint Louis, Missouri; § New York University, College of Arts & Sciences, New York, New York; ¶ University of Florida, Department of Emergency Medicine, Gainesville, Florida; || University of California at San Diego, College of Arts & Sciences, San Diego, California; # Florida State University, College of Medicine, Tallahassee, Florida; ** George Washington University, Washington District of Columbia; †† Tulane University, New Orleans, Louisiana; ‡‡ University of Florida, Department of Radiology, Gainesville, Florida; §§ University of Florida, Department of Surgery, Gainesville, Florida

## Abstract

**Introduction:**

Although there are approximately 1.1 million case presentations of mild traumatic brain injury (mTBI) in the emergency department (ED) each year, little data is available to clinicians to identify patients who are at risk for poor outcomes, including 72-hour ED return after discharge. An understanding of patients at risk for ED return visits during the hyperacute phase following head injury would allow ED providers to develop clinical interventions that reduce its occurrence and improve outcomes.

**Methods:**

This institutional review board-approved consecutive cohort study collected injury and outcome variables on adults with the purpose of identifying positive predictors for 72-hour ED return visits in mTBI patients.

**Results:**

Of 2,787 mTBI patients, 145 (5%) returned unexpectedly to the ED within 72 hours of hospital discharge. Positive predictors for ED return visits included being male (p=0.0298), being black (p=0.0456), having a lower prehospital Glasgow Coma Score (p=0.0335), suffering the injury due to a motor vehicle collision (p=0.0065), or having a bleed on head computed tomography (CT) (p=0.0334). ED return visits were not significantly associated with age, fracture on head CT, or symptomology following head trauma. Patients with return visits most commonly reported post-concussion syndrome (43.1%), pain (18.7%), and recall for further clinical evaluation (14.6%) as the reason for return. Of the 124 patients who returned to the ED within 72 hours, one out of five were admitted to the hospital for further care, with five requiring intensive care unit stays and four undergoing neurosurgery.

**Conclusion:**

Approximately 5% of adult patients who present to the ED for mTBI will return within 72 hours of discharge for further care. Clinicians should identify at-risk individuals during their initial visits and attempt to provide anticipatory guidance when possible.

## INTRODUCTION

Traumatic brain injury (TBI) occurs when an outside force, such as a blow to the head, alters brain function;^1^ it remains a leading cause of injury-related death and disability in developed countries.^2^ In the U.S. alone, TBI accounts for 1.4 million case presentations to the emergency department (ED) annually, with 80% of these cases categorized as mild TBI (mTBI).^3^ Despite its high prevalence, optimal ED management strategies for patients presenting with mTBI remain controversial, and no standardized protocol has been introduced.^3^ Additionally, clinicians make little effort to identify patients at high risk for poor outcomes, such as ED return visits, when designing a treatment plan. Identification of positive predictors for patients at risk of returning to the ED within 72 hours of discharge could lead to improved patient outcomes and conserve hospital resources.

The incidence of unplanned ED return after trauma is not insignificant. Previous estimates of trauma- related ED return visits range from 0.38% to 44%,^4^,^5^ but incidence of unplanned ED return following mTBI has not been reported, even though one of the most common reasons for it is failure to improve after discharge.^6^ More information is needed to understand the underlying causes of unplanned ED return visits in cases of mild mTBI so that clinicians may develop clinical interventions to reduce its occurrence. The goal of this project was to identify factors associated with 72-hour unplanned ED return visits in our mTBI population. A second goal was to investigate complaints upon return, course of treatment, and outcomes for those ED recidivists.

## METHODS

This was an institutional review board-approved retrospective chart review of consecutive adult patients presenting to the ED with mTBI, defined as a Glasgow Coma Scale (GSC) of 13 or greater, during a 43-month period from January 1, 2008, to July 3, 2011. The study was conducted in the ED of a Level I trauma center in the southeastern U.S., which has a catchment area for trauma of over one million.

We performed data abstraction using *a priori* designed data abstraction forms, which paralleled the flow of the information in the health record. Possible answers to data capture were unambiguous, with numerical values defined for each answer. We built in drop-down menus, radio buttons, and range checks to further minimize data entry errors. Data entry personnel were trained on the REDCap (Research Electronic Data Capture) system, which is a secure, web-based application designed to support traditional case report form data capture, and they were blinded to the outcome of interest. We performed statistical analyses using JMP 10 for Macintosh.

Cohort identification was accomplished via identification of ICD-9 codes assigned to head injury, as previously reported by the authors^7^. We classified TBI severity using the Glasgow Coma Score, with GCS 13–15 considered as mild, GCS 9–12 as moderate, and a score less than 9 classified as severe. Post-injury symptomology collected included the occurrence and length of loss of consciousness (LOC), posttraumatic amnesia (PTA), seizure, vomiting, and an alteration of consciousness (AOC). An AOC was defined as being present if the patient reported any of the following: feeling dazed or confused, having difficulty thinking, or if the neurologic exam revealed a decreased mental status.

We also collected data for mechanism of injury, including a fall, motor vehicle collision (MVC), object striking the head, recreational activity, sports, and assault. For patients who returned to the ED within 72 hours of ED discharge, reason(s) for ED return, course of treatment, and outcome were also collected. Two patients had planned 72-hour ED return and were not considered for analysis.

## RESULTS

### Demographics of Mild TBI Cohort

The mTBI cohort consisted of 2,567 patients, of whom 35% were admitted to the hospital, with a median length of stay of two days (IQR 1–4, range 1–59). GCS scores were 13 (3%), 14 (11%), and 15 (86%). Men accounted for 57.5%. One hundred twenty-four (4.8%) returned to the ED unexpectedly within 72 hours of discharge.

### Injury Characteristics of Mild TBI Cohort

Positive loss of consciousness at the time of head injury was reported in 47.8%. Almost one third (27.9%) experienced posttraumatic amnesia for events before and/or after head injury. Altered mental status was experienced by 28.0%. Six percent reported at least one episode of vomiting following head trauma, and 1.8% suffered from seizure after injury. A computed tomography (CT) was performed in 2,347 or 91.4% of the cohort. Of the 2,347 who had CTs, it was abnormal in 27.8% of the cases. Of those with an abnormal CT (n=652), 27.3% or 178 patients had skull or calvarial fracture, and 91.4% or 596 patients had intracranial hemorrhage.

### Demographics of 72-Hour ED Return Cohort

The ED return visit cohort consisted of 124, with 83 being men. Men had a higher median age at 46, compared with 39 years for women. The racial composition was 68% white, 23% black, 6% Hispanic, and 3% other. Fall was the most commonly reported initial mechanism of injury (49%), followed by MVC (34%), and a strike to the head (29%). Seventy percent were transported by EMS, 6% by air and 64% by ground.

### Determinants of Unplanned 72-Hour ED Return

A return ED visit was significantly more common in males (p=0.02), who accounted for 66.9% of this subpopulation. Additionally, patients with an intracranial bleed on head CT were significantly more likely to return to the ED within 72 hours of discharge (p=0.03); 74.5% with 72-hour ED return had intracranial bleed on head CT. Black patients were more likely to return to the ED (p=0.0456). Other predictors included mechanism of MVC (p=0.0065), and a lower prehospital GCS. Among signs and symptoms related to traumatic brain injury, the only symptom that was significant was LOC > 30 min (p=0.0381), of which there were 29 (3%). In contrast, vomiting, seizure, alteration of consciousness and posttraumatic amnesia were not associated with increased risk of ED return visit. A finding of fracture on head CT was not predictive of a patient’s likelihood to return to the ED, nor was the patient’s age.

### Reasons for ED Return

Patients most commonly returned to the ED for symptoms of post-concussion syndrome (46.0%), including headache, altered mental status, and vomiting. Twenty-three patients (18.7%) reported pain and 14.6% were recalled to the ED after discharge for further evaluation, while 9.76% returned for evaluation of a repeat head injury.

### Treatment Course Upon Return to ED

Of the 124 patients who returned to the ED within 72 hours, head CTs were performed in 47 patients, with 17 requiring a hospital stay. Eighty percent of patients were discharged from the ED after treatment, but one out of five was admitted to the hospital for further care. Five of these patients had intensive care unit (ICU) stays (4%), and four (3.2%) required neurosurgery. No in-hospital mortality was reported. One patient left the ED without treatment.

## DISCUSSION

Several studies have recently attempted to characterize factors associated with ED return visits following trauma. Caulfield et al.^5^ found that the rate of ED return visits in men is higher than in women, a finding supported by others.^4^–^8^ One study^9^ reported a higher rate of ED return visit in association with young age and low socioeconomic status, since they are more likely to use the ED as a source of primary medical care.^10^ Meanwhile, another study^11^ found that patients who receive compassionate contact from clinicians are less likely to return to the ED for further care. No studies to the authors’ knowledge, however, focus on the characterization of mTBI return visits.

A 72-hour ED return visit rate of 5% was demonstrated in this study for adult mTBI patients. Additionally, our data confirm that ED return following trauma is not always an unpredictable event, as we found a few descriptors associated with it. Compared to mTBI patients who presented once to the ED, the patients with repeat visits tended to be men, black, have suffered a MVC, and to have a bleed on head CT during the initial ED visit (Table 1). Intracranial bleed complicates initial evaluation of mild head injury since a small percentage of patients with intracranial hemorrhage remain neurologically stable during clinical evaluation but then deteriorate within 24 hours of injury.^12^,^13^ We suspect that the significant rate of return ED visits associated with bleed on head CT is driven by two factors. First, neurological symptoms do not appear immediately with intracranial hemorrhage, so patients may be discharged before clinical assessment can identify anything of medical concern. Second, delayed neurological deterioration encourages individuals to seek further medical care. The best predictor of this progressive intracranial hemorrhage is the male sex,^14^ which perhaps partly explains the male sex as a predictor for 72-hour ED return following mild TBI. Symptomology following head injury, such as loss of consciousness, was also related to risk of ED return visit (Table 1). With the exception of gender and race, the mTBI return visit cohort reflects the demographics of the surrounding population (Table 2).

Four complaints represented 86% of 72-hour ED return visits for the mTBI cohort (Table 3). Post-concussion syndrome was the most common complaint and was reported by nearly half of all patients with return ED visits. Post-concussion syndrome is a term given to describe a variety of physical, cognitive, emotional and sleep symptoms^14^ (Table 4) that arise following head injury. These can be difficult to predict,^15^ although one study suggested that headache and alteration of consciousness immediately following the head injury, and consumption of alcohol prior to it, are predictive.^16^ The second most commonly reported complaint upon ED return was pain, particularly of the back and limbs. Some patients were called back to the ED for further evaluation after receiving test results, while other patients suffered repeat head injuries that required medical attention.

These common complaints allowed us to identify potential areas for improvement to reduce the rate of ED return visits following mTBI. First, it is possible that patient education about post-concussion syndrome could be a successful and economical strategy to reduce ED return visits. If patients expect symptoms such as headache or vomiting after hospital discharge and understand that the majority of patients experience complete resolution of these symptoms within days of onset,^17^ fewer individuals are likely to return to the ED for further evaluation, thus conserving hospital resources and mitigating mTBI’s financial burden on the patient. Second, improved pain management is an opportunity to lower ED return visit rates. Assessment of a patient’s pain prior to discharge could eliminate the immediate need to return for pain management. Third, mTBI patients should not be discharged until imaging studies have been reviewed. This would allow medical personnel to determine if further evaluation is needed while the patient is on site in order to eliminate patient recall to the ED. Fourth, mTBI patients are at heightened risk for head injury compared to the general population,^18^,^19^ signifying that specific discharge instructions that limit return to normal activity could reduce a patient’s risk of recurrent head injury and improve patient outcome.

By identifying at-risk patients for unplanned return visits and following the aforementioned guidelines, we could improve patient outcomes in cases of mTBI. Twenty percent of our return visit cohort was admitted to the hospital upon return to the ED, and these individuals represent two distinct groups: patients whose condition deteriorated after discharge and patients who initially required hospital admission but were overlooked. Identification of at-risk patients could reduce the overlooking of patients requiring hospital admission by encouraging close observation. Of the return visits admitted to the hospital, four patients required ICU says and five underwent neurosurgery (Table 5). This demonstrates that 72-hour ED return can be associated with life-threatening conditions and should not be ignored. Early intervention could improve patient outcomes and reduce the rate of ED return.

## LIMITATIONS

First, this was a single-center study. It is possible that some patients returned to the ED of a surrounding hospital rather than to our study center; therefore, our study likely underestimates the true level of ED return visits following mTBI. Second, our study analyzed positive predictors for return visits within 72 hours of initial discharge. The determinants for ED return during the hyperacute phase after brain injury might not be associated with return visits beyond 72 hours after injury, limiting the study’s generalizability to beyond 72 hours. Future studies should attempt to identify predictors for less immediate ED return after mild TBI as well.

## CONCLUSION

Approximately 5% of adult patients who present to the ED for mild TBI will return within 72 hours of discharge for further care. Predictors of return visits include being male being black, having a lower prehospital GCS score, suffering the injury due to a motor vehicle collision, or having intracranial hemorrhage on CT.

Clinicians should identify at-risk individuals during their initial visits and attempt to provide anticipatory guidance when possible.

## Figures and Tables

**Table 1:** Determinants of unplanned 72-hour ED return for patients with mild traumatic brain injury.

	Unplanned return ED visit – yes (124 patients)	Unplanned return ED visit – no (2,443 patients)	p-value
Age	Mean= 45.9 SD= 22.5	Mean= 43.0 SD= 21.5	0.15
Gender – % male	66.9%	57.0%	0.02
Black race	22%	16%	0.04
Vomiting at time of head trauma	6.4%	6.0%	0.84
Seizure at time of head trauma	3.2%	1.7%	0.22
Loss of consciousness	43.5%	48.0%	0.93
Alteration of consciousness	24.2%	28.2%	0.48
Post traumatic amnesia	28.2%	27.9%	0.99
Fracture on head CT	18.2%	18.1%	0.99
Bleed on head CT	74.5%	60.0%	0.03

*ED*, emergency department; *CT*, computed tomography

**Table 2:** Demographic characteristics of 72-hour return cohort.

Demographic characteristics	n (%)
Race	
White	84 (67.7%)
Black	28 (22.6%)
Hispanic	8 (6.5%)
Native Hawaiian/Pacific Islander	1 (0.8%)
Native American	3 (2.4%)
Gender	
Men	83 (66.9%)
Women	41 (33.1%)
Median age	
Men	46 (IQR 25–57)
Women	39 (IQR 25–79)
Mechanism of injury	
Fall	71 (49%)
Object struck head	44 (29%)
Traffic accident	27 (34%)

**Table 3:** Most common reasons for 72-hour emergency department return.

Reason	Percentage of patients
Post-concussion syndrome	43.1%
Called back for further evaluation	14.6%
Pain	18.7%
Repeat head injury	9.8%

**Table 4:** Signs and symptoms associated with post-concussion syndrome.

Type of symptom following head injury	Signs and symptoms
Physical	Headache
	Nausea
	Vomiting
	Balance problems
	Dizziness
	Visual problems
	Fatigue
	Sensitivity to noise or light
	Numbness or tingling
	Feeling dazed or stunned
Cognitive	Feeling mentally “foggy”
	Feeling mentally slowed down
	Difficulty concentrating
	Difficulty remembering
	Forgetful of recent conversations
	Confused about recent events
	Answers questions slowly
	Repeats questions
Emotional	Irritability
	Sadness
	More emotional
	Nervousness
Sleep	Drowsiness
	Sleeping less than usual
	Sleeping more than usual
	Trouble falling asleep

**Table 5:** Course of treatment for mild traumatic brain injury patients with ED return.

Course of treatment	n (%)
Left without treatment	1 (0.8%)
Discharged from ED	98 (79%)
Admitted to hospital	25 (20.2%)
Computed tomography	47 (37.9%)
ICU stay	5 (4.0%)
Neurosurgery	4 (3.2%)

*ED*, emergency department; *ICU*, intensive care unit
